# Association between the neutrophil-to-lymphocyte ratio and obstructive sleep apnea: a meta-analysis

**DOI:** 10.1038/s41598-020-67708-w

**Published:** 2020-07-02

**Authors:** Min-Seok Rha, Chang-Hoon Kim, Joo-Heon Yoon, Hyung-Ju Cho

**Affiliations:** 1 Department of Otorhinolaryngology, Yonsei University College of Medicine, 50 Yonsei-ro, Seodaemun-gu, Seoul, 03722 Republic of Korea; 2 Graduate School of Medical Science and Engineering, Korea Advanced Institute of Science and Technology, Daejeon, 34141 Republic of Korea; 3 The Airway Mucus Institute, Yonsei University College of Medicine, Severance Hospital, Seoul, 03722 Republic of Korea; 4 Korea Mouse Phenotyping Center, Yonsei University College of Medicine, Seoul, 03722 Republic of Korea

**Keywords:** Sleep disorders, Biomarkers, Biomarkers

## Abstract

This meta-analysis is aimed to investigate the association between the neutrophil-to-lymphocyte ratio (NLR) and obstructive sleep apnea (OSA). The PubMed, Web of Science, Google Scholar, and Cochrane Library databases were searched to collect all relevant articles. The pooled standardized mean difference (SMD) with a 95% confidence interval (CI) was calculated using the random effects model. In addition, subgroup analysis and meta-regression analysis were performed. Eleven eligible articles containing 2,259 patients with OSA were included in this study. Pooled outcomes revealed that the NLR was significantly higher in patients with OSA than in controls (SMD 0.62, 95% CI 0.29–0.94, *P* = 0.002). In subgroup analyses, differences in the NLR between patients and controls increased with worsening OSA grades. Furthermore, meta-regression analysis showed that differences in mean BMI exerted a significant effect on differences in the NLR (*P* = 0.0003). In summary, our meta-analysis demonstrated that the NLR in OSA patients was significantly higher than that in controls, and the difference was larger in patients with severe OSA. These results indicate that the NLR may be a reliable marker for detecting systemic inflammation and predicting disease severity in patients with OSA.

## Introduction

Obstructive sleep apnea (OSA) is a chronic inflammatory disorder characterized by recurrent episodes of partial or complete upper airway obstruction during sleep^1,2^, affecting 3%–9% of the general population^3^. OSA is a serious health problem associated with cardiovascular diseases, neurological diseases, and various types of mortality^2,4–6^. Although the precise underlying mechanisms are not fully understood, systemic inflammation has been proposed as a key factor in the pathogenesis of cardiovascular complications in OSA patients^7,8^. Therefore, investigating the inflammation process is crucial for the management of OSA.

Recently, several markers of systemic inflammation that are obtainable from routine blood tests have attracted attention because of their wide availability and low cost. Among them, the neutrophil-to-lymphocyte ratio (NLR) has been recognized as a reliable measure of systemic inflammation with prognostic value in various chronic diseases^9–12^, likely because chronic systemic inflammation activates white blood cells during disease progression.

Previous studies have investigated the relationship between the NLR and OSA but with inconsistent and controversial results^13–22^. Some researchers have observed significantly elevated NLRs in patients with OSA compared to control groups^13,16–20,22^, whereas others have not^14,15,21^. The incongruity of these results might have been due to multiple factors, including the study design, statistical power of the study, and genetic heterogeneity of the study population. Therefore, we conducted a comprehensive and systematic review and meta-analysis of related studies to determine the associations between the NLR and the presence and severity of OSA.

## Methods

### Search strategy

We conducted this systematic review in adherence to the guidelines of the Preferred Reporting Items for Systematic Reviews and Meta-Analyses^23^. A systematic search of publications in the PubMed, Web of Science, Google Scholar, and Cochrane Library electronic databases (until March 24, 2019) was conducted using the following MeSH terms and keywords: “sleep apnea syndromes”[MeSH], “obstructive sleep apnea”, “sleep apnea”, “OSA”, “sleep-disordered breathing”, “sleep disorders”, “neutrophil to lymphocyte ratio”, “neutrophil lymphocyte ratio”, “neutrophil-to-lymphocyte ratio”, and “NLR”. Supplementary Table 1 describe the full search strategy.

### Eligibility criteria, study selection, and quality assessment

The PICOS (Population, Intervention, Comparator, Outcome and Study design) approach was utilized to define study eligibility. (1) P: adult patients with OSA (defined as apnea–hypopnea index (AHI) of ≥ 5/hour in sleep studies), (2) I: assessment of NLR, (3) C: subjects without OSA or patients with OSA after continuous positive airway pressure (CPAP) treatment (4) O: the NLR value, (5) S: observational studies including cross-sectional studies, cohort studies, case–control studies, or case series. In addition, studies were eligible if they were written in English and full-text publications. Abstracts were independently screened for relevance by two investigators. The full text of relevant articles was reviewed independently by two authors. The references of the retrieved articles were also searched to identify additional studies. Any disagreement between the reviewers was resolved by a third investigator.

The Newcastle–Ottawa scale (NOS) was used to assess the quality of each study based on the following components: selection of the cohort, comparability of cohorts on the basis of the design or analysis, how the exposure was ascertained, and how the outcomes of interest were assessed^24^. Two researchers independently evaluated the quality of each study. Disagreement between the researchers was resolved through consensus. Studies achieving six or more stars were considered to be of high quality.

### Data extraction

The extracted information was as follows: author name, publication year, country, study design, age, sex, mean body mass index (BMI), sample number, OSA severity, and NLR. When the data were only graphically represented^18^, NLR values were extracted from the graph using Adobe Acrobat’s measuring tools (Adobe Systems Incorporated, San Francisco, CA, USA).

### Statistical analysis

The meta-analysis of enrolled studies was performed using R 3.4.3 version statistical software (R Core Team (2017). R: A language and environment for statistical computing. R Foundation for Statistical Computing, Vienna, Austria. URL https://www.R-project.org/.). The difference in the NLR between the OSA and control groups was assessed using the standardized mean difference (SMD). Heterogeneity across the enrolled studies was calculated using the I^2^ test, where I^2^ > 50% indicated significant heterogeneity between studies and prevented reliance on combined study results. In these cases, the random effects model was used to generate pooled effects. By contrast, outcomes without a significant level of heterogeneity (I^2^ < 50) were analyzed using the fixed effects model. Subgroup analysis was performed to evaluate the effects of disease severity, BMI, country, and sex. We also conducted meta-regression analysis to identify possible sources of heterogeneity. Differences in mean age (years), proportion of male patients (%), and mean BMI were the sources of heterogeneity assessed. A funnel plot and Egger test were used to detect publication bias^25^. Additionally, the Duval and Tweedie trim-and-fill method was used to adjust for missing studies and correct the overall effect size according to publication bias^26^. Sensitivity analyses were performed to estimate the influence of each study on the overall meta-analysis results^27^.

## Results

### Literature search and study characteristics

**Figure 1:**
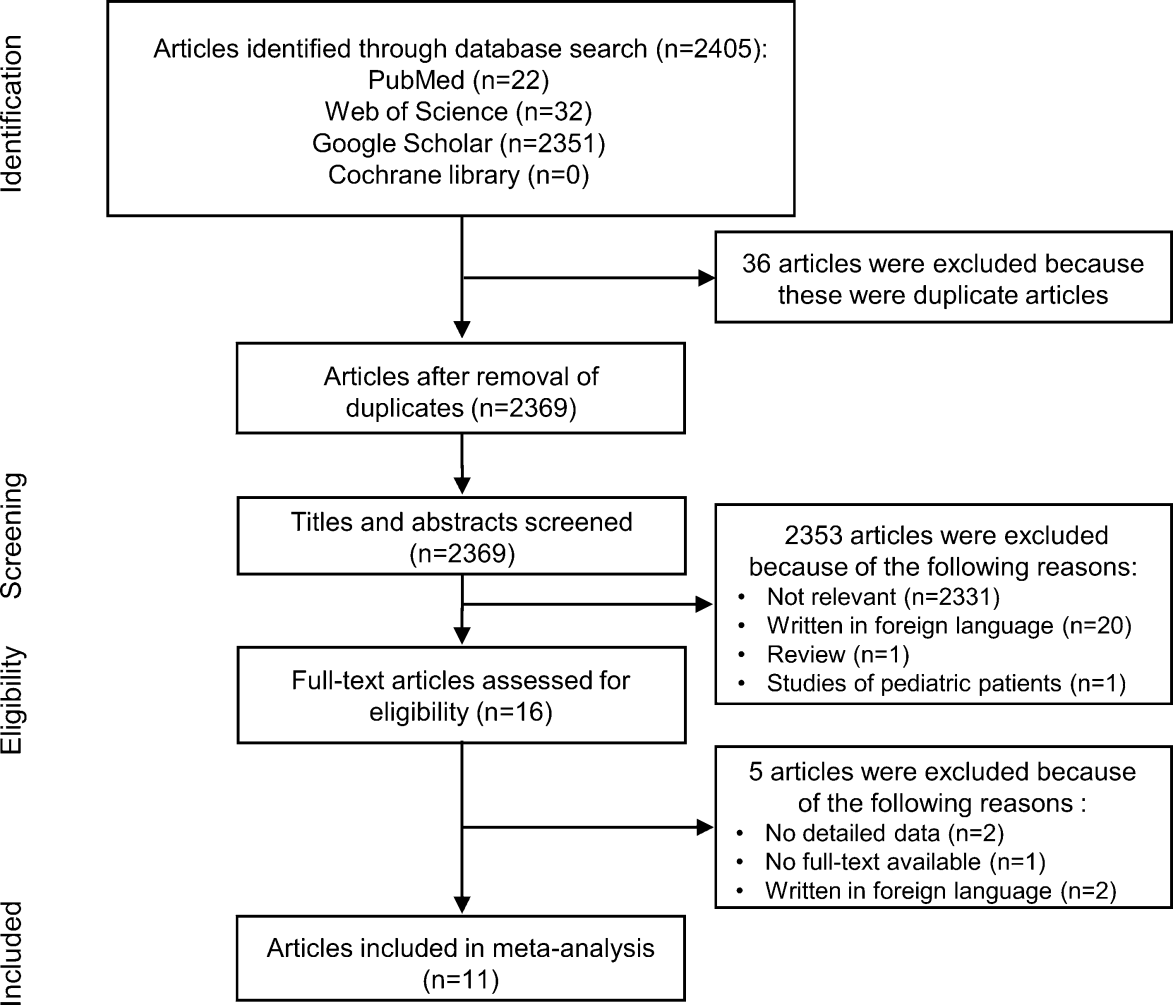
Flow diagram of study search and selection.

The database search using the aforementioned keywords and search strategies returned 2,405 articles, 36 of which were excluded because of duplication. After the title and abstract screening, we excluded 2,353 articles and read the full text of only 16 articles. Finally, 11 articles involving 2,259 OSA patients were included in our meta-analysis^13–22,28^. The process of study selection is presented in Fig. 1. Among the 11 studies, nine were conducted in Turkey and two were conducted in East Asia (Japan and China). The NOS scores of enrolled studies ranged from 6 to 7, and the publication year ranged from 2015 to 2019. All enrolled studies were case series and hospital-based studies. Eight articles^13–15,17–19,21,22^ provided data based on OSA severity (mild: 5 ≤ AHI < 15; moderate: 15 ≤ AHI < 30; and severe: 30 ≤ AHI) and one article^20^ provided data based on BMI (normal weight: BMI < 25; overweight: 25 ≤ BMI ≤ 30; and obesity: 30 < BMI). Two studies^18,28^ provided NLR value before and after CPAP treatment. The characteristics of the enrolled studies are summarized in Table 1.

**Table 1:** Characteristics of enrolled studies.

First author	Year	Country	Study design	OSA severity	OSA					NOS
					n	Age (mean ± SD)	Sex (M/F)	BMI (mean)	NLR (mean ± SD)	
Kivanc^a^	2018	Turkey	RCS	Control	52	41 ± 12	35/17	29	1.90 ± 1.03	6
				Mild	53	43 ± 10	44/10	29	2 ± 1	
				Moderate	65	50 ± 11	47/28	31.3	1.8 ± 1	
				Severe	130	51 ± 11	101/29	34	1.8 ± 0.7	
Fan^a^	2019	China	RCS	Control	135	46.3 ± 12.04	135/0	24.0	1.72 ± 0.74	7
				Mild	185	44.9 ± 11.1	185/0	25.4	1.75 ± 0.95	
				Moderate	171	46.0 ± 11.17	171/0	26.7	1.78 ± 0.67	
				Severe	596	43.5 ± 10.93	596/0	29.1	1.76 ± 0.82	
Korkmaz^a^	2015	Turkey	RCS	Control	40	43.3 ± 11.14	14/26	29.27	1.80 ± 0.64	6
				Mild	27	44.96 ± 9.25	18/9	29.15	1.78 ± 0.57	
				Moderate	37	47.24 ± 9.12	21/16	31.97	1.57 ± 0.54	
				Severe	43	49.35 ± 9.79	26/17	32.6	1.61 ± 0.56	
Yenigun^a^	2015	Turkey	PCS	Control	38	48.08 ± 8.82	20/18	30.54	1.70 ± 0.71	7
				Mild	34	46.75 ± 8.06	24/10	33.97	1.69 ± 0.69	
				Moderate	30	53.64 ± 12.60	14/16	33.53	2.44 ± 1.44	
				Severe	34	52.94 ± 12.21	18/16	36.15	3.37 ± 1.21	
Uygur^a^	2016	Turkey	RCS	Control	118	50.3 ± 11.7	61/57	29.4	1.81 ± 0.50	7
				Mild	57	53.7 ± 10.8	36/21	30.8	2.39 ± 0.60	
				Moderate	53	51.8 ± 12.1	30/23	31.6	3.34 ± 0.90	
				Severe	61	54.5 ± 12.7	39/22	32.1	4.18 ± 1.10	
Oyama^a^	2016	Japan	PCS	Control	5	50 ± 14.6	2/3	21.9	1.43 ± 0.36	7
				Mild	14	61.8 ± 17.3	9/5	22.4	1.66 ± 0.72	
				Moderate	26	63.8 ± 9.4	18/8	26.3	1.94 ± 0.80	
				Severe	50	59.3 ± 13.1	43/7	28.7	2.55 ± 1.62	
				Moderate-severe (pre-CPAP)	29	62.3 ± 9.5	24/5	27.3	1.85 ± 0.71	
				Moderate-severe (post-CPAP)	29	62.3 ± 9.5	24/5	27.3	1.62 ± 0.58	
Sunbul	2015	Turkey	RCS	Control	65	48.7 ± 10.2	42/23	26.9	1.49 ± 0.48	7
				All	130	49.87 ± ND	91/39	34.42	1.87 ± 0.80	
Bozkuş^b^	2018	Turkey	RCS	Control	42	44.02 ± 11.35	25/17	23.31	1.55 ± 0.16	7
				All (normal weight)	36	42 ± 11.24	20/16	23.34	1.59 ± 0.15	
				All (overweight)	38	43 ± 7.16	21/17	27.82	1.83 ± 0.30	
				All (obesity)	39	43.69 ± 7.42	25/14	36.29	2.98 ± 0.29	
Koseoglu^a^	2015	Turkey	RCS	Control	48	43.08 ± 8.88	29/19	27.06	2.02 ± 0.85	6
				Mild	67	ND	ND	ND	1.97 ± 1.25	
				Moderate	61	ND	ND	ND	1.87 ± 0.66	
				Severe	108	ND	ND	ND	1.85 ± 0.64	
Günbatar^a^	2015	Turkey	RCS	Control	26	44.7 ± 10.4	21/5	27.15	1.73 ± 0.68	6
				Moderate	22	49.3 ± 10.6	16/6	32.4	1.95 ± 0.90	
				Severe	63	51.02 ± 11.1	50/13	33.5	2.29 ± 1.40	
Özdemir	2019	Turkey	PCS	Moderate-severe (pre-CPAP)	29	46 ± 10.11	18/11	34.85	1.56 ± 0.47	6
				Moderate-severe (post-CPAP)	29	46 ± 10.11	18/11	35.51	1.63 ± 0.58	

*OSA* obstructive sleep apnea, *NOS* Newcastle–Ottawa scale, *PCS* prospective case series, *RCS* retrospective case series, *ND* not determined, *SD* standard deviation, *BMI* body mass index, *NLR* neutrophil-to-lymphocyte ratio, *M* male, *F* female.

^a^These articles divided patients into three groups according to disease severity (mild: 5 ≤ apnea–hypopnea index (AHI) < 15; moderate: 15 ≤ AHI < 30; and severe: 30 ≤ AHI).

^b^This article divided patients into three groups according to body mass index (BMI) (normal weight: BMI < 25; overweight: 25 ≤ BMI ≤ 30; and obesity: 30 < BMI).

### Associations between the NLR and the presence of OSA

**Figure 2:**
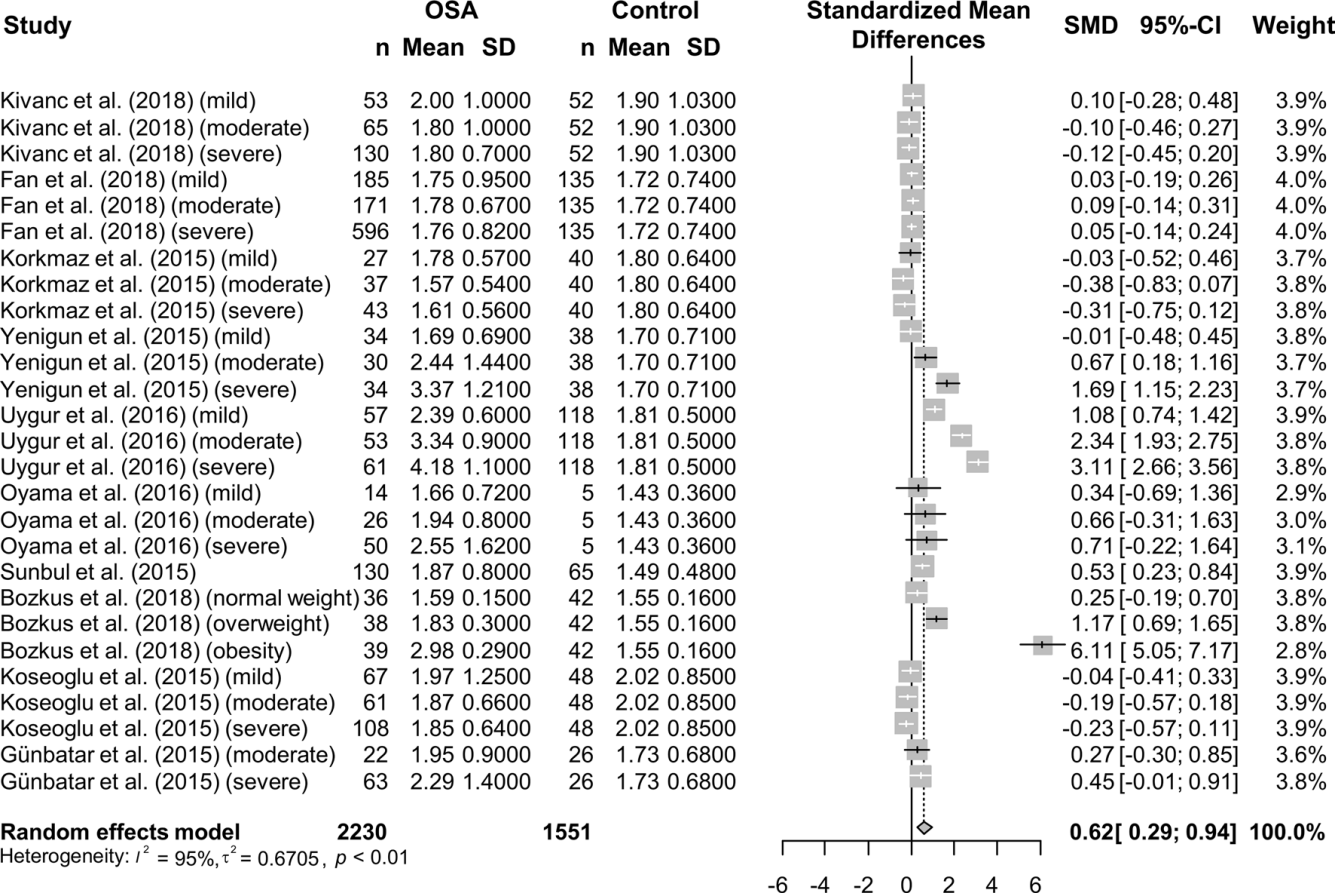
Comparison of the neutrophil-to-lymphocyte ratio between obstructive sleep apnea patients and controls. Calculation was based on the random effects model. The results were expressed as the standardized mean difference and 95% confidence intervals. OSA, obstructive sleep apnea; SD, standard deviation; SMD, standardized mean difference; CI, confidence interval.

Since 10 studies^13–22^ fully provided NLR values of control subjects and patients with OSA, we included those studies for the meta-analysis to investigate association between NLR and the presence of OSA. We used the random effects model due to significant heterogeneity between the enrolled studies (I^2^ = 95%). A significant difference in the NLR was observed between the OSA and control groups (SMD = 0.62, 95% confidence interval (CI) = 0.29–0.94, *P* = 0.002) (Fig. 2).

### Subgroup analysis

**Figure 3:**
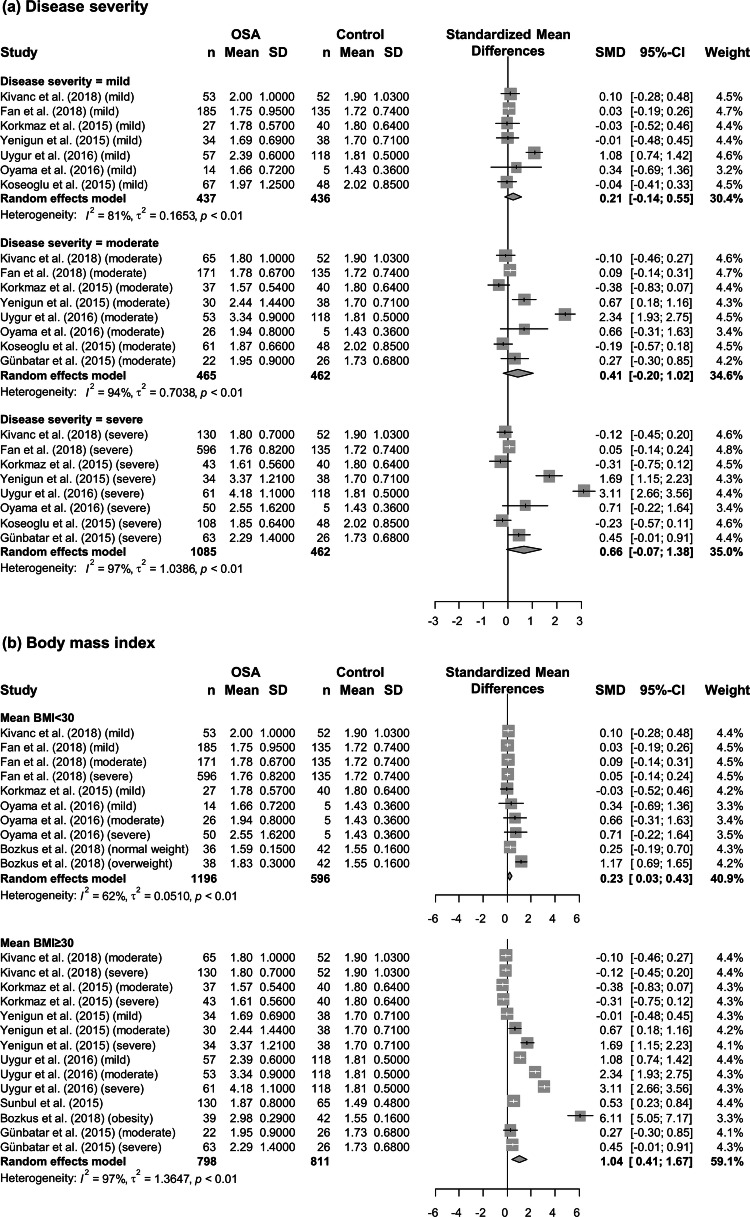
Subgroup analysis based on disease severity evaluated using the apnea–hypopnea index and body mass index. (**a**) Studies were divided into three subgroups (mild: 5 ≤ apnea–hypopnea index (AHI) < 15; moderate: 15 ≤ AHI < 30; and severe: 30 ≤ AHI) according to disease severity. (**b**) Studies were divided into two subgroups (body mass index (BMI) < 30 and BMI ≥ 30) according to the mean BMI of patients. Calculation for each subgroup was based on the random effects model. The results were expressed as the standardized mean difference and 95% confidence intervals. OSA, obstructive sleep apnea; SD, standard deviation; SMD, standardized mean difference; CI, confidence interval; BMI, body mass index.

**Figure 4:**
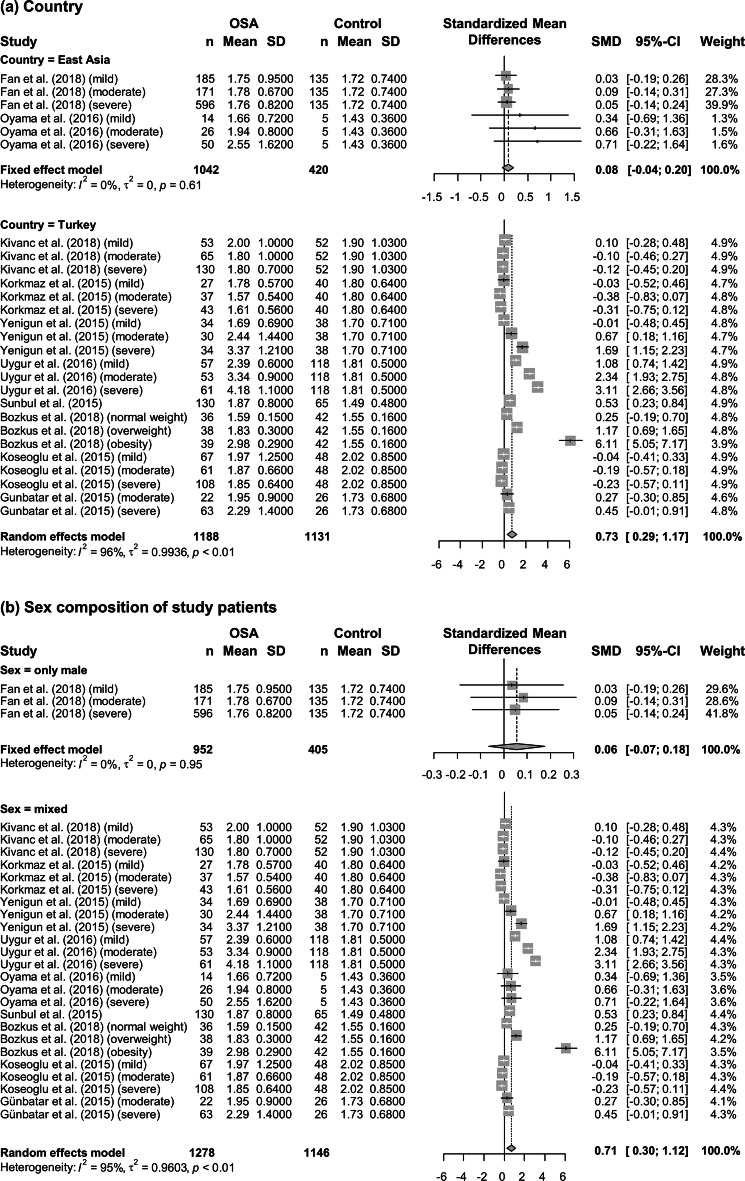
Subgroup analysis based on country and sex composition of study patients. (**a**) Studies were divided into two subgroups (East Asia and Turkey) according to the country in which the research was conducted. Calculation for studies from East Asia and Turkey was based on the fixed and random effects models, respectively. (**b**) Studies were divided into two subgroups (male and mixed sex) according to the sex composition of the study patients. Calculation for studies with only male patient groups and studies with mixed-sex patient groups was based on the fixed and random effects models, respectively. The results were expressed as the standardized mean difference and 95% confidence intervals. OSA, obstructive sleep apnea; SD, standard deviation; SMD, standardized mean difference; CI, confidence interval.

To identify possible sources of heterogeneity, subgroup analysis was performed according to the severity of OSA, BMI, country, and sex. When studies were stratified by disease severity, differences in the NLR between patients and controls increased gradually with OSA severity. The NLR in patients with mild, moderate, and severe OSA was significantly higher than that in controls by 0.21 (95% CI = − 0.14 to 0.55, *P* < 0.01), 0.41 (95% CI = − 0.20 to 1.02, *P* < 0.01), and 0.66 (95% CI = − 0.07 to 1.38, *P* < 0.01), respectively (Fig. 3a). Furthermore, both non-obese (BMI < 30; SMD = 0.23, 95% CI = 0.03–0.43, *P* < 0.01) and obese patients (BMI ≥ 30; SMD =  1.04, 95% CI = 0.41–1.67, *P* < 0.01) showed significantly higher NLR compared to controls (Fig. 3b). In subsequent subgroup analysis according to country, the difference in the NLR between patients and controls was not statistically significant in studies from East Asia (SMD = 0.08, 95% CI = − 0.04 to 0.20, *P* = 0.61), whereas it was statistically significant in studies from Turkey (SMD = 0.73, 95% CI = 0.29–1.17, *P* < 0.01) (Fig. 4a). When we analyzed data according to the sex composition of study patient groups, a statistically significant difference in the NLR between patients and controls was observed in studies with mixed sex patient groups (SMD = 0.71, 95% CI = 0.30–1.12, *P* < 0.01), but not in studies with only male patients (SMD = 0.06, 95% CI = − 0.07 to 0.18, *P* = 0.95) (Fig. 4b).

### Meta-regression analysis

We conducted a meta-regression analysis to further assess the effect of confounding factors on differences in the NLR between OSA patients and controls. The outcome variable was the SMD of the NLR, and the covariates included differences in age (years), BMI, and the proportion of male patients (%). Only the difference in BMI (*P* = 0.0003) was found to exert a significant effect on the difference in the NLR, whereas differences in age (*P* = 0.3849) and the proportion of male patients (%) (*P* = 0.1275) showed no significant effect (Supplementary Fig. 1a–c).

### Publication bias

Visual inspection of funnel plots revealed the existence of asymmetry (Supplementary Fig. 2). Egger’s regression test also suggested evidence of publication bias (*t* = 2.2406, *P* = 0.0372). However, the trim-and-fill method showed that no study required to ensure symmetry and the adjusted SMD was not changed (adjusted SMD = 0.62, 95% CI 0.29–0.94, *P* = 0.002).

### Sensitivity analysis

Stability of the results was evaluated through sensitivity analysis. The corresponding pooled SMD values were not substantially altered when single studies were sequentially removed, with an effect size ranging between 0.46 and 0.66, suggesting that the results of the meta-analysis were stable (Supplementary Fig. 3).

### The effect of CPAP treatment on NLR

In our primary search results, two studies^18,28^ compared NLR before and after CPAP treatment. We performed a meta-analysis of those studies to investigate the effect of CPAP treatment on NLR. There was no significant difference in NLR before and after treatment with CPAP (*P* = 0.20) (Supplementary Fig. 4).

## Discussion

Although the association between the NLR and OSA has drawn considerable attention and has been widely investigated, previous results have been controversial. In the present study, we comprehensively analyzed the results of 10 studies through a meta-analysis and found that the NLR value was higher in OSA patients than in control subjects. These results indicate that neutrophilic inflammation may play a key role in the pathogenesis of OSA.

In recent years, both local and systemic inflammation as well as changes in immune response have been reported in patients with OSA. Previous studies have indicated that intermittent hypoxia selectively activates nuclear factor-κB (NF‑κB) pathways^29,30^. In addition, the levels of several inflammatory markers, including C-reactive protein, interleukin-6, and tumor necrosis factor, have been widely recognized as being higher in OSA patients^31–33^, suggesting that inflammatory activation is critical in the pathogenesis of OSA. CPAP treatment significantly reduces elevated inflammatory markers in OSA patients^34^. Several studies have also demonstrated that systemic inflammation may contribute to a higher risk of cardiovascular diseases in patients with OSA^7,35^ and elevated levels of inflammatory markers are associated with a higher cardiovascular risk^36^.

Because neutrophils and lymphocytes play a major role in inflammatory responses through the release of various inflammatory mediators, their absolute counts or relative values may reflect the status of systemic inflammation. NLR is superior to other individual leukocyte parameters (e.g. neutrophil, lymphocyte, and total leukocyte counts) in terms of stability, because it is a ratio of two different immune pathways^37,38^. Hence, the NLR has been proposed as a novel inflammatory marker associated with many chronic diseases, including coronary heart disease, hypertension, pulmonary thromboembolism, psoriasis, and various malignancies^12,39–41^. A previous study also reported an association between an elevated NLR and mortality in a group of non-ST-elevation myocardial infarction patients^42^. Similarly, our meta-analysis demonstrated that the NLR was associated with OSA. However, NLR may be influenced by certain factors, including bacterial infection^43^ and medications^44,45^. Therefore, the NLR is a particularly reliable marker in patients without conditions that may affect inflammatory response. The mechanism underlying the elevation of the NLR in OSA patients may be related to hypoxia-induced chronic inflammation. Neutrophils from patients with moderate and severe OSA demonstrate prolonged survival, which is associated with higher NF‑κB levels and worse balance of pro-apoptotic and anti-apoptotic proteins^30,46,47^. In the case of lymphocytes, some researchers have proposed that the decrease in lymphocytes reflects a higher degree of physiological stress^48,49^.

In subgroup analyses, we found that the difference in the NLR between patients and controls increased gradually with the severity of OSA. These findings strongly suggest that the NLR may reflect disease severity, and the activation of the inflammatory process is linked to the disease activity of OSA. In addition, considering that the difference in the NLR between patients and controls was larger in obese patients and the difference in BMI was correlated with the NLR in meta-regression analysis, obesity may play a key role in systemic inflammation in OSA patients via unknown mechanisms. Furthermore, several investigators reported that the NLR value was higher in obese individuals than in control subjects with normal weight^50,51^. Therefore, comparison with BMI-matched controls is needed to avoid the confounding effect of obesity on NLR. Notably, no statistically significant differences in the NLR were observed ​​between patients and controls in studies with only male patients and studies from East Asia. Previous studies using a large US national data set reported that females and non-Hispanic black were associated with lower NLR^52,53^, indicating that gender and ethnicity may affect NLR value. In addition, given that racial difference in inflammatory responses has been proposed^54^, increase of inflammatory biomarker levels in patients with OSA may be different between races. Similarly, differential inflammatory responses between male and female subjects might explain the discrepancy in the results among each subgroup according to sex composition. It has been described that females show a more pronounced response to inflammatory stimuli^55^. Furthermore, pro-inflammatory cytokine responses and NF-κB activation of mononuclear cells following sleep disturbance were more exaggerated in females than in males^56,57^. However, concluding a relationship between these parameters and the NLR in OSA patients may be premature because the results were derived from few studies. Future studies with a large number of patients may be required to obtain more definitive results.

This study had several limitations. First, there might be a referral bias because all the studies enrolled in this meta-analysis were hospital-based studies. Population-based studies may provide more accurate information on the general population, compared to hospital-based studies. Second, we excluded articles in foreign languages, which may have biased the results. Third, the medication status and co-morbidities of OSA patients were unknown, which may represent uncontrollable factors in our analysis. Fourth, all enrolled studies were case series, which generally have a lower level of evidence than prospective cohort studies. Fifth, a potential bias may be derived from the geographical background of included studies. The majority of included studies investigated Turkish population. Further studies conducted in other countries are required to validate our results. Despite these limitations, this meta-analysis has several considerable strengths. First, we included the largest number of studies available. Second, our study was the first to conduct analyses to investigate the effects of potential modifiers.

In summary, our meta-analysis demonstrated that the NLR of patients with OSA was significantly higher than that of controls. Furthermore, the difference in the NLR between patients and controls gradually increased with the severity of OSA. These findings indicate that the NLR may be a reliable marker for detecting systemic inflammation and predicting disease activity in patients with OSA.

## Supplementary information

Supplementary Information
