# Supplementary material for: Association between the neutrophil-to-lymphocyte ratio and obstructive sleep apnea: a meta-analysis
Source: Sci Rep. 2020 Jul 2;10:10862. doi: 10.1038/s41598-020-67708-w (PMC7331605; doi:10.1038/s41598-020-67708-w)
Supplement: Supplementary file 1 — Supplementary Information [file 41598_2020_67708_MOESM1_ESM.pdf]

Supplementary materials for

Association between the Neutrophil-to-Lymphocyte Ratio and Obstructive Sleep Apnea: A Meta-analysis

Min-Seok Rha, Chang-Hoon Kim, Joo-Heon Yoon, Hyung-Ju Cho

Supplementary Table 1. Search strategy

Source	Search Terms
Pubmed	(“sleep apnea syndromes”[MeSH] OR “obstructive sleep apnea”[All Fields] OR “sleep apnea”[All Fields] OR OSA[All Fields] OR “sleep-disordered breathing”[All Fields] OR “sleep disorders”[All Fields]) AND (“neutrophil to lymphocyte ratio”[All Fields] OR “neutrophil lymphocyte ratio”[All Fields] OR “neutrophil-to-lymphocyte ratio”[All Fields] OR NLR[All Fields])
Web of Science	ALL=((“obstructive sleep apnea” OR “sleep apnea” OR OSA OR “sleep-disordered breathing” OR “sleep disorders”) AND (“neutrophil to lymphocyte ratio” OR “neutrophil lymphocyte ratio” OR “neutrophil-to-lymphocyte ratio” OR NLR))
Google Scholar	(“obstructive sleep apnea” OR “sleep apnea” OR OSA OR “sleep-disordered breathing” OR “sleep disorders”) AND (“neutrophil to lymphocyte ratio” OR “neutrophil lymphocyte ratio” OR “neutrophil-to-lymphocyte ratio” OR NLR)
The Cochrane Library	#1: (“obstructive sleep apnea” OR “sleep apnea” OR OSA OR “sleep-disordered breathing” OR “sleep disorders”)<br>#2: MeSH descriptor: [sleep apnea syndromes] explode all trees<br>#3: (“neutrophil to lymphocyte ratio” OR “neutrophil lymphocyte ratio” OR “neutrophil-to-lymphocyte ratio” OR NLR)<br>#4: #1 or #2<br>#5: #3 and #4

Supplementary Figures Legends

Supplementary Figure 1. Meta-regression analysis.

Bubble plots of the associations between SMD of NLR and each parameter. (a) difference in mean age (years), (b) difference in mean BMI, (c) difference in proportion of male patients (%). Solid line represents inverse variance-weighted meta-regression trend line. BMI, body mass index; SMD, standardized mean difference.

Supplementary Figure 2. The funnel plot for exploring the publication bias.

SMD, standardized mean difference.

Supplementary Figure 3. Sensitivity analysis of the meta-analysis.

A forest plot for the sensitivity analysis is presented. Calculation was based on the random effects model. The results were expressed as the standardized mean difference and 95% confidence intervals. SMD, standardized mean difference; CI, confidence interval.

Supplementary Figure 4. Comparison of the neutrophil-to-lymphocyte ratio before and after treatment with CPAP.

Calculation was based on the fixed effect model. The results were expressed as the standardized mean difference and 95% confidence intervals. CPAP, continuous positive airway pressure; SD, standard deviation; SMD, standardized mean difference; CI, confidence interval.

Supplementary Figure 1

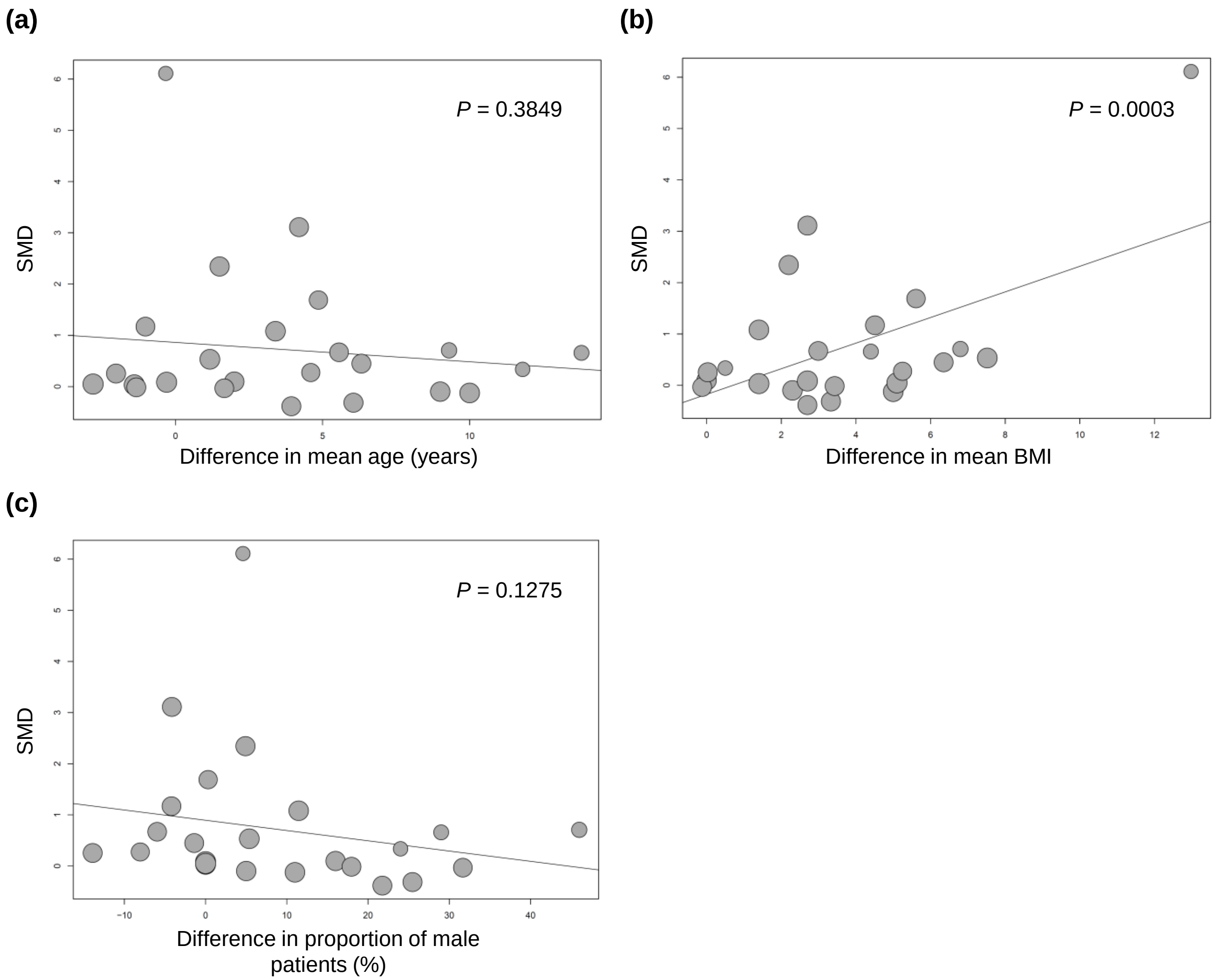

Supplementary Figure 2

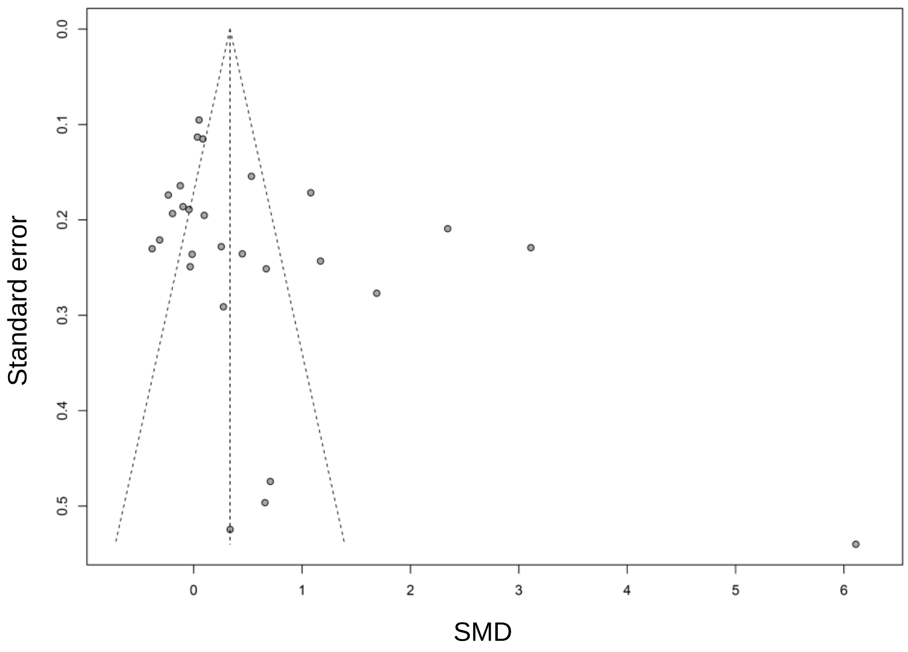

Supplementary Figure 3

Study	SMD	95%-CI
Omitting Kivanc et al. (2018) (mild)	0.64	[0.30; 0.98]
Omitting Kivanc et al. (2018) (moderate)	0.65	[0.31; 0.98]
Omitting Kivanc et al. (2018) (severe)	0.65	[0.31; 0.99]
Omitting Fan et al. (2018) (mild)	0.65	[0.30; 0.99]
Omitting Fan et al. (2018) (moderate)	0.64	[0.30; 0.99]
Omitting Fan et al. (2018) (severe)	0.65	[0.30; 1.00]
Omitting Korkmaz et al. (2015) (mild)	0.64	[0.31; 0.98]
Omitting Korkmaz et al. (2015) (moderate)	0.66	[0.33; 0.99]
Omitting Korkmaz et al. (2015) (severe)	0.66	[0.32; 0.99]
Omitting Yenigun et al. (2015) (mild)	0.64	[0.31; 0.98]
Omitting Yenigun et al. (2015) (moderate)	0.62	[0.28; 0.95]
Omitting Yenigun et al. (2015) (severe)	0.58	[0.25; 0.90]
Omitting Uygur et al. (2016) (mild)	0.60	[0.27; 0.93]
Omitting Uygur et al. (2016) (moderate)	0.54	[0.24; 0.84]
Omitting Uygur et al. (2016) (severe)	0.50	[0.22; 0.78]
Omitting Oyama et al. (2016) (mild)	0.63	[0.30; 0.96]
Omitting Oyama et al. (2016) (moderate)	0.62	[0.29; 0.95]
Omitting Oyama et al. (2016) (severe)	0.62	[0.29; 0.95]
Omitting Sunbul et al. (2015)	0.62	[0.28; 0.96]
Omitting Bozkus et al. (2018) (normal weight)	0.63	[0.30; 0.97]
Omitting Bozkus et al. (2018) (overweight)	0.60	[0.27; 0.93]
Omitting Bozkus et al. (2018) (obesity)	0.46	[0.16; 0.75]
Omitting Koseoglu et al. (2015) (mild)	0.65	[0.31; 0.98]
Omitting Koseoglu et al. (2015) (moderate)	0.65	[0.32; 0.99]
Omitting Koseoglu et al. (2015) (severe)	0.65	[0.32; 0.99]
Omitting Günbatar et al. (2015) (moderate)	0.63	[0.30; 0.97]
Omitting Günbatar et al. (2015) (severe)	0.63	[0.29; 0.96]
**Random effects model**	**0.62**	**[0.29; 0.94]**

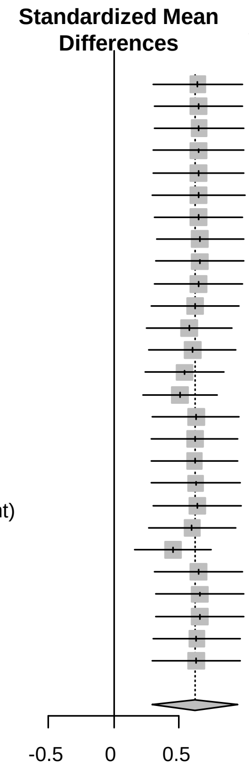

Supplementary Figure 4

Study	Post-CPAP n	Post-CPAP Mean	Post-CPAP SD	Pre-CPAP n	Pre-CPAP Mean	Pre-CPAP SD	Standardized Mean Differences	SMD	95%-CI	Weight
Oyama et al.	29	1.62	0.5800	29	1.85	0.7100		−0.35	[−0.87; 0.17]	49.6%
Ozdemir et al.	29	1.63	0.5800	29	1.56	0.4700		0.13	[−0.38; 0.65]	50.4%
**Fixed effect model**	**58**			**58**				**−0.11**	**[−0.47; 0.26]**	**100.0%**

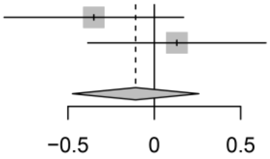

Heterogeneity: $I^2 = 40\%$, $\tau^2 = 0.0460$, $p = 0.20$
